# A potential new pathway for heparin treatment of sepsis-induced lung injury: inhibition of pulmonary endothelial cell pyroptosis by blocking hMGB1-LPS-induced caspase-11 activation

**DOI:** 10.3389/fcimb.2022.984835

**Published:** 2022-09-15

**Authors:** Rui Yang, Xiaojuan Zhang

**Affiliations:** Department of Critical Care Medicine, the First Hospital of China Medical University, Shenyang, China

**Keywords:** acute lung injury, pyroptosis, endothelial cells, high mobility group 1, caspase-11, heparin

## Abstract

Sepsis is a significant cause of mortality in critically ill patients. Acute lung injury (ALI) is a leading cause of death in these patients. Endothelial cells exposed to the bacterial endotoxin lipopolysaccharide (LPS) can progress into pyroptosis, a programmed lysis of cell death triggered by inflammatory caspases. It is characterized by lytic cell death induced by the binding of intracellular LPS to caspases 4/5 in human cells and caspase-11 in mouse cells. In mice,caspase-11-dependent pyroptosis plays an important role in endotoxemia. HMGB1 released into the plasma binds to LPS and is internalized into lysosomes in endothelial cells *via* the advanced glycation end product receptor. In the acidic lysosomal environment, HMGB1 permeates the phospholipid bilayer, which is followed by the leakage of LPS into the cytoplasm and the activation of caspase-11. Heparin is an anticoagulant widely applied in the treatment of thrombotic disease. Previous studies have found that heparin could block caspase-11-dependent inflammatory reactions, decrease sepsis-related mortality, and reduce ALI, independent of its anticoagulant activity. Heparin or modified heparin with no anticoagulant property could inhibit the alarmin HMGB1-LPS interactions, minimize LPS entry into the cytoplasm, and thus blocking caspase-11 activation. Heparin has been studied in septic ALI, but the regulatory mechanism of pulmonary endothelial cell pyroptosis is still unclear. In this paper, we discuss the potential novel role of heparin in the treatment of septic ALI from the unique mechanism of pulmonary endothelial cell pyroptosis.

## Introduction

Sepsis is a significant cause of mortality. Acute lung injury (ALI) is a leading cause of death in critically ill patients with sepsis. Acute respiratory distress syndrome (ARDS) is the most serious form of ALI (Ashbaugh et al., 1967; Matthay et al., 2019). Its in-hospital mortality rate is approximately 35% to 46% (Bellani et al., 2016; Sauer et al., 2021; Peukert et al., 2021). During sepsis, infiltration of inflammatory cells in the lung can lead to irreversible damage, which finally develops into ALI or even ARDS (Kumar and Chhibber, 2011). Both direct lung epithelial injury and indirect endothelial cell injury can cause sepsis-related ALI/ARDS (Englert et al., 2019; Huppert et al., 2019). Pro-inflammatory cytokines can activate the pulmonary endothelial cells with subsequent increased expressions of e-selectin or endothelium-endothelial adhesion molecule-1 (ELam-1), or leucocyte adhesion molecule-2 (lecMA-2), which further induces neutrophil infiltration. This, together with the extensive endothelial cell death and disintegration of endothelial adhesion junctions, can contribute to pulmonary endothelial barrier disruption and the development of ALI (Mehta and Malik, 2006; Gong et al., 2015; Matthay et al., 2019).

Recent studies suggested that the caspase-11 signaling pathway participates in the pathogenesis of sepsis (Kayagaki et al., 2011; Kayagaki et al., 2015; Cheng et al., 2017; Deng et al., 2018). Caspase-11 can be found in various cell types, including pulmonary endothelial cells and macrophages (Cheng et al., 2017; Deng et al., 2018). Caspase-11 activated by the intracellular endotoxin lipopolysaccharide (LPS) cleaves Gasdermin D (GSDMD) into polypeptides to form a nanopore on the cytoplasmic membrane (Kayagaki et al., 2015; He et al., 2015; Shi et al., 2015; Ding et al., 2016). This process not only results in a lysis form of programmed cell death named pyroptosis but also activates living cells to secret interleukin (IL)-1 (He et al., 2015; Ding et al., 2016; Zanoni et al., 2016; Evavold et al., 2018). ECs demonstrated lytic cell death, activation of Gsdmd, and release of the proinflammatory cytokine IL-1β, processes that are dependent on caspase-4/5 in human ECs and caspase-11 in mouse Ecs (Cheng et al., 2017).

Activations of caspase-11 and GSDMD are facilitated by high mobility group box 1 (HMGB1). HMGB1 is a 25 kD nuclear protein found in all cell types and is highly conserved in mammals. The intracellular functions of HMGB1 include gene transcription and chromatin repair regulation (Müller et al., 2001; Lotze and Tracey, 2005; Davalos et al., 2013). Extracellular HMGB1 carries the characteristic alarm protein functions to activate innate immunity, and HMGB1 can be actively or passively released after cell death during endotoxemia or sepsis. During an infection, LPS triggers hepatocytes to release HMGB1 into the circulation (Deng et al., 2018). The plasma HMGB1 attaches to extracellular LPS to form advanced glycation end products (RAGE) in the cytoplasm, which induces lysosome rupture by receptor-mediated internalization of LPS. HMGB1 then causes lysosomal rupture and ultimately activates cleavage of caspase-11 and GSDMD (Deng et al., 2018). Animal studies have shown that either deficiency in caspase-11, loss of GSDMD, neutralizing extracellular HMGB1, or deletion of HMGB1 in hepatocytes can improve survival in experimental septic models (Cheng et al., 2017; Deng et al., 2018).

Heparin was discovered in 1916 and is traditionally used as an anticoagulant in the treatment of thrombotic diseases, such as various venous thromboembolism (Hirsh and Levine, 1992; Li and Ma, 2017). Heparin can bind to the lysine residues in antithrombin and induce irreversible conformational changes in arginine reaction sites (Rezaie et al., 2004; Yang et al., 2004), which leads to a more than 100-fold increase in antithrombin activity (Chuang et al., 2001; Rezaie et al., 2004). In addition to its anticoagulant function, heparin was found to have other properties, such as protease modulation and anti-complement and anti-inflammatory activities (Davidson et al., 2002; Hoppensteadt et al., 2008). Heparin therapy has been shown to improve the prognosis of sepsis and alleviate lung injury by inhibiting caspase-11 signaling (Cornet et al., 2007; Li et al., 2011; Liu et al., 2014; Wang et al., 2014; Zarychanski et al., 2015; Fan et al., 2016; Li and Ma, 2017; Liu et al., 2019; Tang et al., 2021). This article proposes a mechanism for how heparin alleviates sepsis-induced ALI from the perspective of pulmonary endothelial cell pyroptosis.

### Endothelial pyroptosis underlies LPS-induced ALI

The pathophysiology of lung injury mainly includes inflammation disorder and increased permeability of the lung endothelium and epithelium. In ARDS, the endothelium permeability is increased allowing movement of fluid and protein through the pulmonary vascular endothelium, leading to interstitial edema. Increased permeability of neutrophils and red blood cells (causing them to accumulate in the alveolar space) is a hallmark of ARDS (Matthay et al., 2019; Lindsey et al., 2019; Gerber et al., 2020). Cardiac outflow goes directly into the lungs and the pulmonary tissues. As a result, pulmonary endothelial cells are constantly the subject of injury from circulating pathogens and bacterial endotoxins such as LPS. This is essential for the development of ALI (Maniatis and Orfanos, 2008). Pulmonary vascular permeability and leukocyte recruitment are maintained by an intact endothelial barrier (Mehta and Malik, 2006; Nourshargh and Alon, 2014). Thus, disruption of the endothelial barrier can lead to pre-clotting pathway activation, pro-inflammatory cytokine release (such as IL-1β), neutrophil influx, and tissue edema (Mehta and Malik, 2006). During inflammation, there is an adaptive increase in endothelial permeability increase to allow white blood cell chemotaxis into the lung to overcome the infection. Severe uncontrolled infection can have massive inflammatory reactions and endothelial barrier destruction, which eventually leads to death in patients with septic ALI. Recent evidence suggests that endothelial injury might be related to pyroptosis of endothelial cells (Cheng et al., 2017).

Pyroptosis, apoptosis, and necrosis are different types of programmed cell death. Each type of programmed cell death is regulated by a unique individual set of host proteins that mediate multiple biological outcomes (Vanden Berghe et al., 2014; Blander, 2014; Chan et al., 2015; Wallach et al., 2016; Man and Kanneganti, 2016; Vande Walle and Lamkanfi, 2016; Weinlich et al., 2017). Caspase activation participates in both apoptosis and pyroptosis. Apoptosis starts with initiator caspases-2, -8, -9, and -10 and ends with executioner caspases-3, -6, and -7 (Fink and Cookson, 2005). Different from apoptosis, pyroptosis is an inflammatory caspase-induced form of necrosis and inflammatory programmed cell death (Vande Walle and Lamkanfi, 2016). Pyroptosis is also distinct from another necrotic and inflammatory form of programmed cell death called necroptosis, because the execution of pyroptosis requires inflammatory caspases (Vercammen et al., 1998; Holler et al., 2000; Weinlich et al., 2017). The role of inflammatory caspases-4/5/11 in the cytoplasmic LPS signal transduction pathway and pyroptosis has been studied mostly in dendritic cells and macrophages (Miao et al., 2011; Kayagaki et al., 2013; Aachoui et al., 2013; Kayagaki et al., 2015; Shi et al., 2015; Zanoni et al., 2016). Furthermore,endothelial cells could have highly sensitive and complex intracellular LPS induction mechanisms that could lead to caspase-4/5/11-dependent endothelial lysis (Shi et al., 2014; Shi et al., 2015). Characteristic presentations include plasma membrane rupture and LDH release, maturation, release of Il-1β, and cleavage of perforin GSDMD by constriction effects (Kayagaki et al., 2015; Shi et al., 2015; Ding et al., 2016; Liu et al., 2016), which causes extensive pulmonary endothelial death and damages the endothelial barrier to induce ALI.

## Mechanism of endothelial cell pyroptosis

### Caspase-11 mediates pyroptosis

In Gram-negative bacterial infections, bacterial lysis leads to septic cascades that release large amounts of endotoxin (LPS) into the circulation (Baumgartner et al., 1985). The typical mechanism by which mammalian host cells detect LPS is achieved by Toll-like receptor 4 (TLR4) on the cell surface (Medzhitov et al., 1997; Poltorak et al., 1998). However, recent studies in macrophages suggested that another LPS-mediated intracellular pathway might play a role in the development of septic shock, namely triggering pyroptosis (Kayagaki et al., 2013; Hagar et al., 2013). The endotoxin LPS could damage the cell membrane and bind to caspase-4/5/11 to activate macrophage pyroptosis, leading to rapid cell lysis (Kayagaki et al., 2013; Hagar et al., 2013; Kayagaki et al., 2015; Jorgensen and Miao, 2015).

Caspase-11 is an intracellular LPS receptor that mediates pyroptosis. Sepsis-related mortality induced by endotoxemia is largely due to caspase-11 activation (Wang et al., 1998; Kayagaki et al., 2011; Kayagaki et al., 2013; Hagar et al., 2013; Kayagaki et al., 2015), which can cleave GSDMD to unleash active membrane pore-forming peptides. The latter can cause cell pyroptosis and release the leukotriene-like LTB4 *via* cyclooxygenase-1 and Alarmins, including IL-1α (Hagar et al., 2013). Studies have shown improved survival in endotoxemia mice with Gasdmd deletion or cyclooxyg (Kayagaki et al., 2013; Hagar et al., 2013; Kayagaki et al., 2015; Jorgensen and Miao, 2015)enase-1 (COX-1) inhibition (Hagar et al., 2013; Kayagaki et al., 2015). Caspase-11-mediated pyroptosis could trigger the local immune defense and disrupt intracellular niches by promoting vascular permeability and releasing chemokines (Kayagaki et al., 2011; Kayagaki et al., 2013; Aachoui et al., 2013; Hagar et al., 2013; Shi et al., 2014).

**Figure 1:**
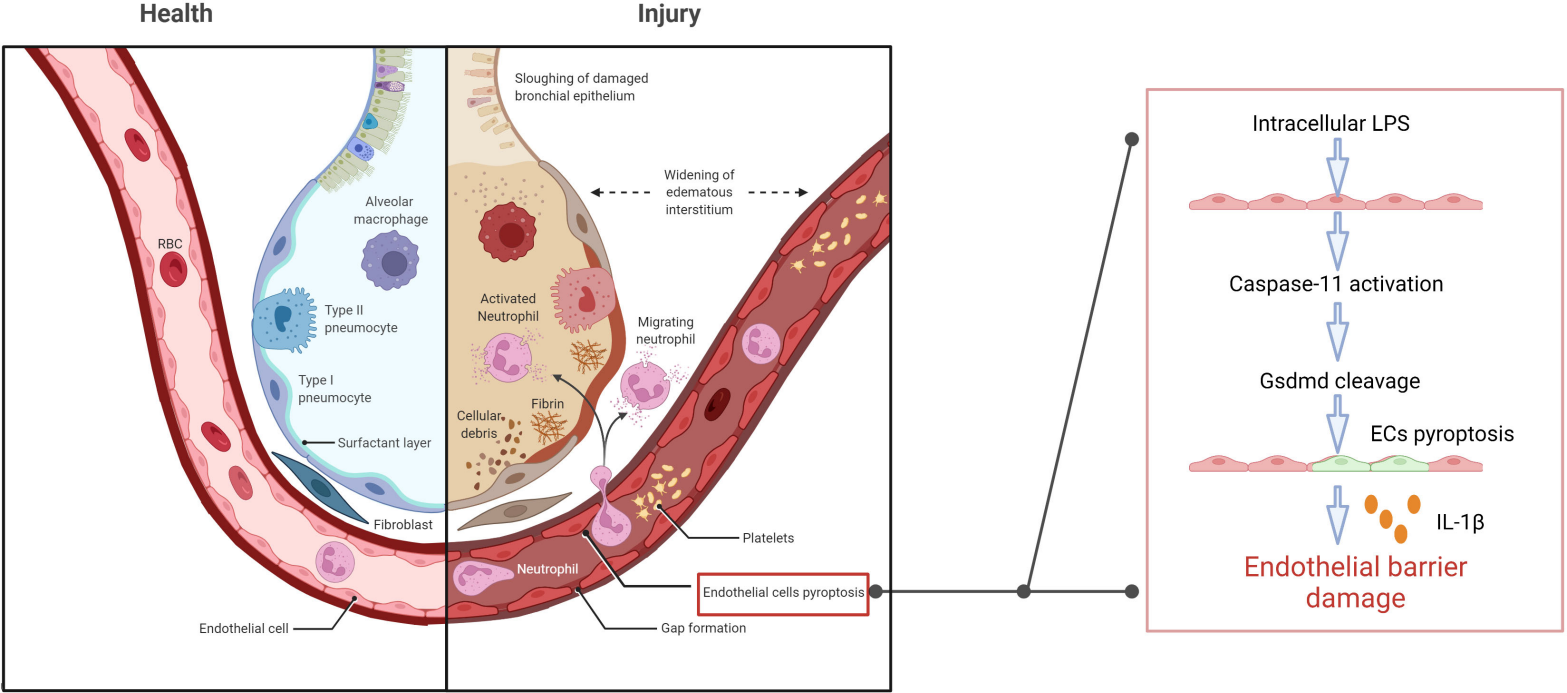
Extracellular LPS enters the endothelial cytoplasm through the cell membrane, and then triggers caspase-11-dependent endothelial cell pyroptosis and damages the pulmonary endothelial barrier, resulting in pulmonary edema, proinflammatory cytokine release, fluid and protein leakage, and massive influx of leukocytes.

Caspase-11-dependent lysis of GSDMD and subsequent release of active membrane pore-forming peptides results in cell swelling, lysis, and death (Shi et al., 2015; Jorgensen and Miao, 2015; Broz, 2015; de Gassart and Martinon, 2015; Ding et al., 2016; Liu et al., 2016; Sborgi et al., 2016; Aglietti et al., 2016; Yuan et al., 2016). Microvesicles containing LPS could be shed from the gram-negative bacteria and fuse with the host cellular membrane to release LPS intracellularly and activate inflammatory cysteine signaling and cell pyroptosis (Vanaja et al., 2016; Man et al., 2016). In mice, endothelial cell pyroptosis is characterized by the caspase-11-dependent cell lysis, GSDMD activation, and release of pro-inflammatory cytokine IL-1β. LPS enters the endothelial cytoplasm through the bacterial microvesicles or destructed inner cortical membrane, subsequently activating caspase-4/5/11 to trigger cell pyroptosis through GSDMD lysis. Extensive endothelial cell lysis results in massive destruction of the pulmonary endothelial barrier (Cheng et al., 2017). Pro-inflammatory cytokine release, leukocyte influx, and pulmonary edema emerge as characteristic signs of ALI (Aird, 2003; Andonegui et al., 2003; Matthay et al., 2019) (**Figure 1**). Interestingly, although endothelial cells could also undergo pyroptosis without activated caspase-11 expression, this required long-term exposure to LPS (Cheng et al., 2017).

Extracellular LPS could trigger pyroptosis of endothelial cells and immune cells only after LPS binds to intracellular caspase-11 (Kayagaki et al., 2013; Aachoui et al., 2013; Hagar et al., 2013; Cheng et al., 2017). Activation of extracellular LPS enhances the expression of caspase-11 in mouse endothelial cells and similarly enhances the expression of caspase-4 and -5 in human endothelial cells, which has been shown to increase the binding of intracellular LPS to these inflammatory caspases (Cheng et al., 2017).

### HMGB1 activates caspase - 11

HMGB1 is named due to its high electrophoretic mobility in an agarose and polyacrylamide gel (Klune et al., 2008). It is a highly conserved and unanimously expressed nuclear protein that participates in the nucleosome structure maintenance and the regulation process of DNA replication, recombination, transcription, and repair (Travers, 2003). HMGB1 is released extracellularly after cell activation, injury, stress, or death and functions as a transporter to promote inflammation (Andersson et al., 2018; Qu et al., 2019). In 1999, it was first reported that HMGB1 could act as a proinflammatory cytokine to stimulate macrophages, which were activated by LPS in sepsis (Wang et al., 1999). Subsequently, it has been reported that HMGB1 could be actively secreted by natural killer cells, monocytes, platelets, endothelial cells, and dendritic cells, and passively released from the nuclei of damaged/necrotic cells during infection. Studies have shown that, after LPS stimulation, HMGB1 was secreted into the extracellular space where it bound to its receptors, including TLR2, TLR4, and RAGE, to induce the expressions of cytokines, adhesion molecules, and chemokines, which further exacerbated the inflammation and injury (Wolfson et al., 2011; Qu et al., 2019). Deletion of the HMGB1 gene or neutralizing circulation of HMGB1 has been shown to have a protective effect against fatal endotoxemia and sepsis (Wang et al., 1999; Wang et al., 2004; Qin et al., 2006; Rittirsch et al., 2008; Lamkanfi et al., 2010; Andersson and Tracey, 2011).

During the signal cascade in sepsis, HMGB1 utilizes a variety of membrane receptors. Its bindings to RAGE and TLR4 occur at its residues 150-183 and 89-108, respectively (Huttunen et al., 2002; Yang et al., 2010). Bioactivity of HMGB1 depends on the redox state of its three cysteine residues (Kazama et al., 2008; Lu et al., 2012). The disulfide isoforms could activate cytokine production and TLR4, while the fully reduced isoforms could not (Lu et al., 2012).

**Figure 2:**
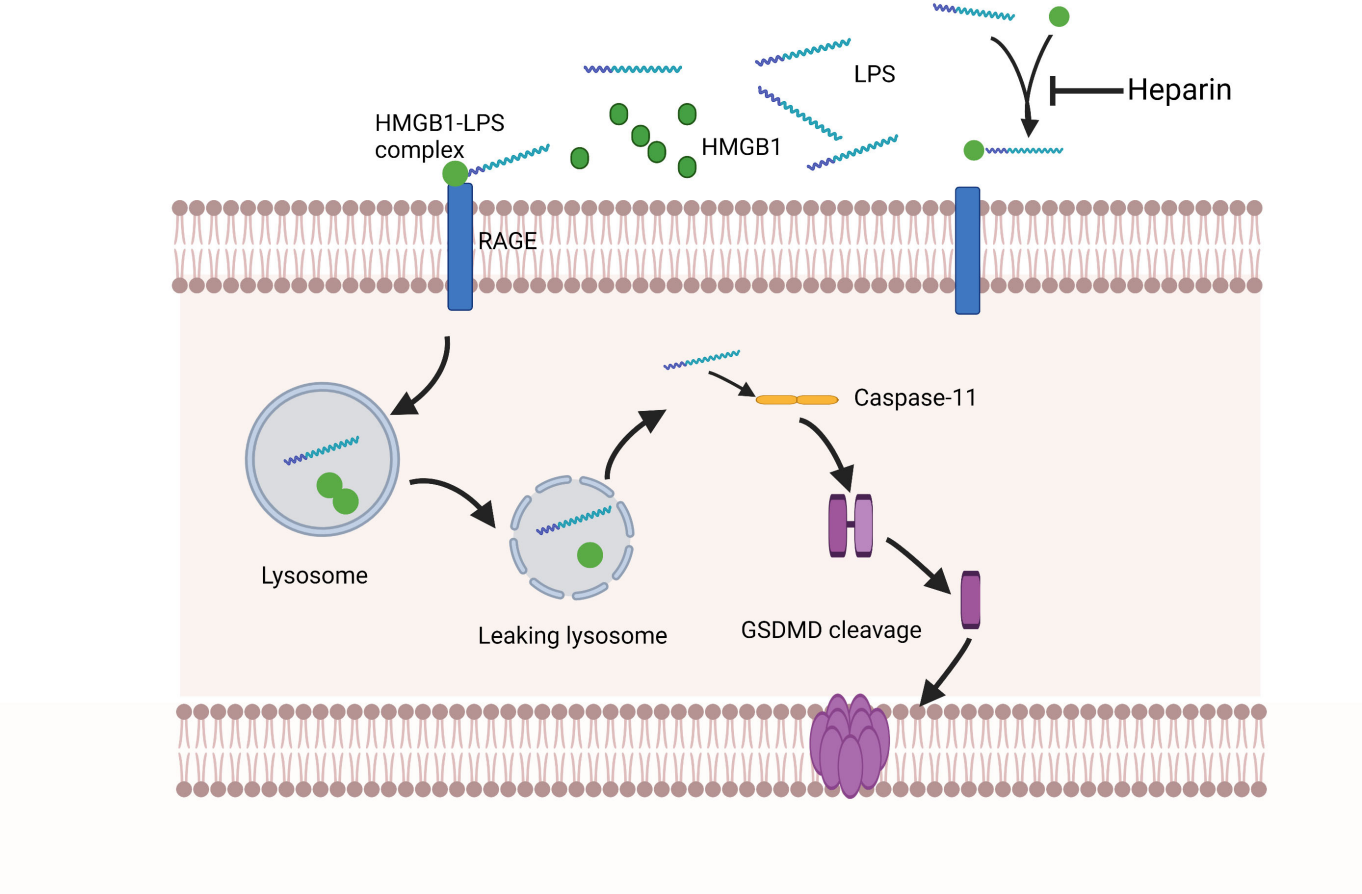
Extracellular HMGB1 and LPS form complexes. These complexes bind to the RAGE expressed by pulmonary endothelial cells, which is followed by the internalization of the HMGB1-LPS complex into lysosomes. Under acidic conditions, HMGB1 acts as a detergent to destroy the lysosome membrane, allowing LPS to enter the cytoplasm and activate caspase-11. Subsequently, activated caspase-11 mediates GSDMD lysis to form pores on the cell membrane, ultimately leading to pulmonary endothelial cell pyroptosis. Heparin has a high affinity for HMGB1, which can compete with the formation of HMGB1-LPS complexes and prevent pulmonary endothelial cell pyroptosis upstream of caspase-11 activation.

Recent studies have shown that LPS relies on HMGB1 and RAGE to assist cell transportation. These molecules could facilitate LPS leakage into the cytoplasm with subsequent activation of the key receptor caspase-11 (Deng et al., 2018). In sepsis, the circulating pathogen-associated molecular patterns (PAMPs), including LPS, can trigger hepatocytes to release HMGB1 into the bloodstream. Extracellular LPS can physically bind to HMGB1, and the HMGB1-LPS complex can be internalized into lysosomes within endothelial cells and macrophages *via* RAGE, disrupting lysosome stability *via* HMGB1 (Deng et al., 2018). HMGB1 has a unique ability to act as a detergent in the lysosomal membranes owing to the acidic conditions of the lysosomal environment (Xu et al., 2014; Deng et al., 2018; Yuan et al., 2020). Thus, partner molecules transported by HMGB1 will avoid the expected degradation in the lysosome and instead leak into the cytoplasm, reaching homologous cytoplasmic receptors and potentiating the pro-inflammatory response. HMGB1 then penetrates directly into the phospholipid bilayer of the lysosome. Using the liposome leakage test and whole-cell patch clamp analysis, it was found that the phospholipid bilayer penetrating ability of HMGB1 was enhanced under acidic conditions, demonstrating the pH-dependent ability of HMGB1 to induce lysosomal rupture in organelles. In addition, it also explained the reason why HMGB1 does not cause cytoplasm or nuclear membrane damages under normal physiological conditions (Deng et al., 2018). These effects of HMGB1 in the lysosome result in the leakage of LPS into the cytoplasm and activation of caspase-11 and pulmonary endothelial cell pyroptosis (**Figure 2**).

Both myeloid and endothelial cells can express RAGE and caspase-11 (Hofmann et al., 1999; Liliensiek et al., 2004). Endothelial caspase-11 participates in pyroptosis induced by endotoxemia (Cheng et al., 2017). Reduced HMGB1 release from hepatocytes, RAGE deficiency, and extracellular HMGB1 neutralization might prevent caspase-11-dependent pyroptosis during endotoxemia and bacterial sepsis (Deng et al., 2018). Interestingly, another study reported that HMGB1 alone could activate ASC-dependent pyroptosis, which was independent of the caspase-11 pathway (Yuan et al., 2020). HMGB1 could also damage the lysosomes and activate the NLRP3 inflammasome independently from the caspase-11 pathway in the macrophages with TLR agonist primers. This response could be the result of HMGB1 oxidation that is followed by the intramolecular bond formation between cysteine 23 and cysteine 45, which causes TLR4-MD2-mediated HMGB1 mobilization to function as a trigger for NLRP3 inflammasome activation (Hornung et al., 2008; Yang et al., 2015; Frank et al., 2016). However, in other studies, HMGB1 alone could not induce pyroptosis. The different redox states of the recombinant HMGB1 protein in these studies might explain the different experimental results (Deng et al., 2018).

### Heparin prevents pyroptosis of EC

Previous studies have found that heparin could attenuate the caspase-11-dependent inflammatory reactions and reduce mortality from sepsis in mice. Both heparin administration and caspase-11 deletion have been shown to reduce lung injury in mice (Tang et al., 2021). Most ligands bind to heparin/heparan sulfate through electrostatic interactions of the positively charged arginine and lysine residues in the ligand with the negatively charged uronic acids and sulfate groups in heparin (Xu et al., 2011). HMGB1 has many basic residues: eight arginine and 43 lysine residues, which altogether account for 24% of HMGB1 amino acids. Among them, six basic residues are important for heparan sulfate binding (Xu et al., 2011). Arg97, Lys87, Lys88, Lys90, and Lys96 residues are within the latter part of the long ring segment that connects A- and B-boxes. As a separate residue on the last spiral of the B-box, Lys150 also participates in heparin-ligand binding (Xu et al., 2011). Heparin can bind to HMGB1 with a high affinity and induce spatial conformational changes in the latter (Ling et al., 2011). These conformational changes in HMGB1 might lower its binding affinity to its receptors. In addition, some studies demonstrated that HMGB1 alone would not cause inflammatory reactions. Purified rHMGB1 could only induce limited cytokine secretions (Rouhiainen et al., 2007; Sha et al., 2008; Youn et al., 2008; Hreggvidsdottir et al., 2009). HMGB1 binds with other mediators, such as IL-1B, DNA, LPS, or nucleosomes, to form complexes associated with inflammation (Sha et al., 2008). HMGB1 also has the allosteric effects to change the conformation of cytokines or their interactions with cytokine receptors (Bianchi, 2009). Tang et al. found that heparin could bind to HMGB1 and block hMGB1-LPS interactions (Tang et al., 2021). In a mouse model of sepsis, it was found that heparin intervention could minimize IL-1α and IL-1β release, as well as GSDMD distributions in the lungs after LPS stimulation (Tang et al., 2021). Meanwhile, in a clinical study of 20 sepsis patients who received heparin and 21 sepsis patients who did not, activation of caspase-4 (the human homologue of caspase-11) was measured (Tang et al., 2021). Markers of caspase-4 activation, including serum levels of IL-1α and IL-1β, were significantly lower in patients who received the heparin treatment compared to those who did not (Tang et al., 2021). RAGE has been shown to be highly expressed in endothelial cells and binds to HMGB1 and other ligands, such as AGEs, S100 proteins, and amyloid proteins, *via* its two N-terminal IgG-like domains (Basta, 2008; Yan et al., 2010). RAGE has also been reported to bind to heparin (Xu et al., 2011), but this mechanism needs to be further investigated. In another study, heparin sulfate, a chemically modified heparin, did not demonstrate any anticoagulant properties, indicating that heparin sulfate could dose-dependently block caspase-11 as a non-anticoagulant heparin (NAH) (Tang et al., 2021). As such, heparin may block caspase-11 by a mechanism independent of its anticoagulant properties, by which it can inhibit pyroptosis of pulmonary endothelial cells and ameliorate ALI during the sepsis (**Figure 2**).

## Conclusion and summary

In pulmonary endothelial cells, there is a highly sensitive and complex intracellular LPS-sensing mechanism that leads to caspase-4/5/11 dependent pyroptosis (Cheng et al., 2017), which is characterized by plasma membrane rupture and LDH release, IL-1β maturation and secretion, and division of constriction-effector perforin GSDMD (Shi et al., 2014; Kayagaki et al., 2015; Shi et al., 2015; Ding et al., 2016). Pyroptosis is an effective method of eliminating the intracellular bacterial niche and releasing inflammatory mediators such as IL-1β, while retaining uninfected adjacent cells (Aachoui et al., 2013; Hagar et al., 2013; Maltez et al., 2015; Jorgensen et al., 2016). Therefore, the innate immune response induced by caspases-1/4/5/11 is different from the typical inflammasome activation pathway mediated through the cell surface TLR4 (Kayagaki et al., 2011; Miao et al., 2011; Kayagaki et al., 2013; Man and Kanneganti, 2016; Broz and Dixit, 2016). On the other hand, In the study of mouse sepsis model, caspase-11 may also be immunopathologic (Napier et al., 2016). Caspase-11 can be activated when LPS is delivered intracellularly into the target cells, such as endothelial cells and macrophages, by HMGB1 secreted by hepatocytes or microvesicles released by bacteria (Vanaja et al., 2016; Deng et al., 2018). This leads to immune cell death with subsequent endothelial barrier disruptions (Vanaja et al., 2016; Cheng et al., 2017; Deng et al., 2018). Endothelial cell-specific deletion of caspase-11 was shown to ameliorate LPS-dependent pulmonary vascular endothelial permeability and significantly improve survival in both endotoxemia models and cecal ligation and puncture-induced ALI models of multi-microbial sepsis (Cheng et al., 2017). Activation of extracellular LPS enhances the expression of caspase-11 in mouse endothelial cells and similarly enhances the expression of caspase-4 and -5 in human endothelial cells (Cheng et al., 2017).

Heparin treatment has been found to block caspase-11-dependent inflammatory reactions and is an effective inhibitor in the caspase-11 pathway during sepsis (Tang et al., 2021). In animal models of fatal endotoxemia or severe sepsis, heparin inhibited the LPS cytoplasmic transmission by blocking the hMGB1-LPS interactions and decreasing the heparinase-induced glycocalygeal degradation in macrophages, thereby attenuating the overactivation of this harmful cascade (Tang et al., 2021). In the mouse experiment,it was found that heparin could block caspase-11 by a mechanism unrelated to its anticoagulant activity, thereby inhibiting cell pyroptosis (Tang et al., 2021). Meanwhile, in the clinical study, markers of caspase-4 activation, including serum levels of IL-1α and IL-1β, were significantly lower in patients who received the heparin treatment compared to those who did not (Tang et al., 2021). A study has also showed that nonanticoagulant heparin, purified from clinical grade heparin, binds histones and prevents histone-mediated cytotoxicity *in vitro* and reduces mortality from sterile inflammation and sepsis in mouse models without increasing the risk of bleeding (Wildhagen et al., 2014). As such this evidence suggests that low dose heparin or modified heparin without anticoagulant properties could be used to achieve a therapeutic effect and reduce the risk of bleeding. This will mean that we find a solution to the conflict between heparin treatment of sepsis ALI and its side effects. This is the purpose of this review.However, other studies show that heparin treatment does not significantly reduce the 28-day mortality of septic patients (Jaimes et al., 2009; Li and Ma, 2017). The discrepancy between these studies might be due to the difference in infected pathogens.Gram-positive sepsis causes death through mechanisms distinct from that of Gram-negative sepsis (Popescu et al., 2018).Thus,further research is needed to better understand this potential role of heparin in treatment of sepsis.

## Author contributions

RY: collected and analyzed the data; drafted the article. XZ: revised and submitted the manuscript. All authors have read and approved the final version of manuscript.

## Funding

The present review was supported by the Liaoning Natural Science Foundation (grant No. 2021-MS-188).

## Acknowledgments

**Figures 1** and **2** were created using BioRender.com.

## Conflict of interest

The authors declare that the research was conducted in the absence of any commercial or financial relationships that could be construed as a potential conflict of interest.

## Publisher’s note

All claims expressed in this article are solely those of the authors and do not necessarily represent those of their affiliated organizations, or those of the publisher, the editors and the reviewers. Any product that may be evaluated in this article, or claim that may be made by its manufacturer, is not guaranteed or endorsed by the publisher.
